# The Three-Herb Formula Shuang-Huang-Lian stabilizes mast cells through activation of mitochondrial calcium uniporter

**DOI:** 10.1038/srep38736

**Published:** 2017-01-03

**Authors:** Yuan Gao, Rui Hou, Qiaoling Fei, Lei Fang, Yixin Han, Runlan Cai, Cheng Peng, Yun Qi

**Affiliations:** 1 Institute of Medicinal Plant Development, Chinese Academy of Medical Sciences & Peking Union Medical College, Beijing, 100193, China; 2 Chengdu University of Traditional Chinese Medicine, Chengdu, 610075, China

## Abstract

Mast cells (MCs) are key effector cells of IgE-FcεRI- or MrgprX2-mediated signaling event. Shuang-Huang-Lian (SHL), a herbal formula from Chinese Pharmacopoeia, has been clinically used in type I hypersensitivity. Our previous study demonstrated that SHL exerted a non-negligible effect on MC stabilization. Herein, we sought to elucidate the molecular mechanisms of the prominent anti-allergic ability of SHL. MrgprX2- and IgE-FcεRI-mediated MC activation *in vitro* and *in vivo* models were developed by using compound 48/80 (C48/80) and shrimp tropomyosin (ST), respectively. Our data showed that SHL markedly dampened C48/80- or ST-induced MC degranulation *in vitro* and *in vivo*. Mechanistic study indicated that cytosolic Ca^2+^ (Ca^2+^_[c]_) level decreased rapidly and sustainably after SHL treatment, and then returned to homeostasis when SHL was withdrawn. Moreover, SHL decreases Ca^2+^_[c]_ levels mainly through enhancing the mitochondrial Ca^2+^ (Ca^2+^_[m]_) uptake. After genetically silencing or pharmacologic inhibiting mitochondrial calcium uniporter (MCU), the effect of SHL on the Ca^2+^_[c]_ level and MC degranulation was significantly weakened. Simultaneously, the activation of SHL on Ca^2+^_[m]_ uptake was completely lost. Collectively, by activating MCU, SHL decreases Ca^2+^_[c]_ level to stabilize MCs, thus exerting a remarkable anti-allergic activity, which could have considerable influences on clinical practice and research.

Mast cells (MCs) originate from the haematopoietic progenitor cells that enter nearly all vascularized tissue, where they complete their maturation and, under some circumstances, can then migrate into epithelia^1^,^2^,^3^. As tissue-resident cells, MCs are strategically situated at host-environment interfaces, such as the skin, respiratory and gastrointestinal tracts, ready to respond to immunogenic stimuli^4^, indicating that they act as key contributors of innate and adaptive immune responses^5^,^6^. MCs are activated on IgE receptor (FcεRI) crosslinking, resulting in the release of a diverse array of preformed cytoplasmic granule-associated mediators (e.g., histamine and β-hexosaminidase, etc.), as well as newly synthesized proinflammatory lipid mediators, cytokines and chemokines^7^,^8^. In the FcεRI-independent pathways, MCs may be activated by numerous stimuli including basic secretagogues [e.g., substance P, compound 48/80 (C48/80) and mastoparan], peptidergic drugs (e.g., icatibant), THIQ motif drugs (e.g., atracurium) and fluoroquinolone family antibiotics (e.g., ciprofloxacin). Recently research revealed that they are all ligands of MrgprX2, an orthologue of the human G-protein coupled mas-related gene receptor^9^,^10^. But whichsoever, IgE-FcεRI- or MrgprX2-mediated MC signaling event, eventually results in the activation of protein kinase C (PKC) and the release of Ca^2+^ from the endoplasmic reticulum (ER), which in turn induces the stromal interaction molecule 1-mediated opening of the store-operated Ca^2+^ channel ORAI1 and then leads to the influx of extracellular Ca^2+^. The influx of Ca^2+^ is amplified by short transient potential Ca^2+^ channel 1. The increase in intracellular Ca^2+^ levels and the activation of PKC trigger MC degranulation^10^,^11^.

Thus, calcium mobilization is a critical event to the activation of MCs and intracellular Ca^2+^ pools are the determining factors of MC degranulation^12^. Mitochondrial Ca^2+^ (Ca^2+^_[m]_) uptake is considered to buffer local or bulk cytosolic Ca^2+^ (Ca^2+^_[c]_) rises^13^. But until recently, the uniporter’s veil began to be lifted. Now, it is known that the uniporter is a multi-subunit Ca^2+^ channel, with the Ca^2+^ pore formed by mitochondrial calcium uniporter (MCU) protein^14^,^15^ and accessory proteins, including MICU1^16^, MICU2^17^, MCUb^18^, MCUR1^19^ and EMRE^20^. Although the precise roles of these accessory proteins is far from clear, they are required either for the channel activity or for regulating MCU under various conditions. MCU, an approximate 40-kDa protein, possesses two predicted transmembrane domains, which forms (through oligomerization) a gated ion channel^21^. Mutation of a single amino acid (serine 259) resulted in a uniporter that loses the ability to be deactivated by the classical inhibitor ruthenium red. Moreover, mutations in the acidic linker domain resulted in markedly diminished calcium uptake^22^. The fact that mitochondria buffer the Ca^2+^_[c]_ rises by accumulating Ca^2+^ into their matrix raises the question whether the activating MCU may dampen MC degranulation for the treatment of allergy, anaphylaxis and asthma, etc.

Shuang-Huang-Lian (SHL), a formula containing *Lonicerae Japonicae Flos, Scutellariae Radix* and *Fructus Forsythiae*, is consistently prepared by stringent manufacturing procedure from Chinese Pharmacopoeia^23^. Clinically, SHL products, generally considered as the antimicrobial agent, are delivered through different routes (e.g., oral, injectable and pulmonary routes, etc.)^23^,^24^, and widely used to treat upper respiratory tract infection, pneumonia, tonsillitis, and other respiratory diseases caused by bacterium/viruses^25^. Our previous studies indicated that SHL protected lung tissue from infections via the potential anti-inflammatory and anti-oxidative activities^25^,^26^. In addition, SHL has also been applied in the type I hypersensitivity, including bronchial asthma^27^,^28^,^29^, vernal keratitis^30^, urticaria and eczema^31^, by using the aerosol inhalation or intravenous drip. Indeed, the excellent MC stabilization effect of SHL was observed. The present study focused on the underlying molecular mechanism of SHL. Our findings reveal that, for the first time, SHL potently stabilizes MCs through decreasing Ca^2+^_[c]_ level by activating MCU independent of Ca^2+^_[c]_ rise, which is different from the conventional MC stabilizers (e.g., cromolyn sodium and ketotifen).

## Results

### SHL exerts prominent effects on C48/80-induced MC activation *in vitro* and *in vivo*

**Figure 1:**
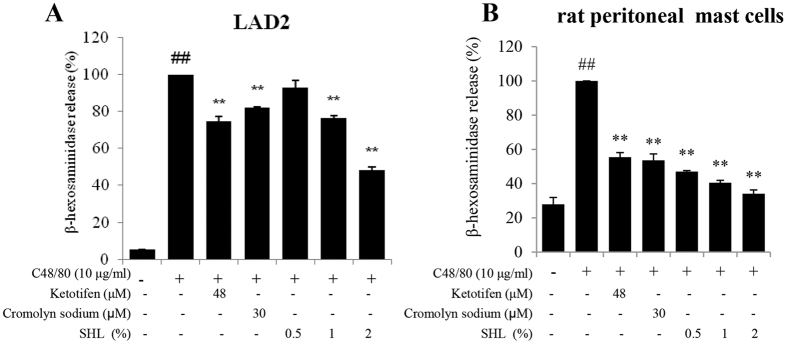
SHL suppressed C48/80-induced β-hexosaminidase releases *in vitro*. The LAD2 cells **(A)** and rat peritoneal MCs **(B)** were stimulated by C48/80 with or without SHL. β-hexosaminidase release was determined 1.5 h after C48/80 challenge. Ketotifen and cromolyn sodium were used as a positive control. ^##^*P* < 0.01 *vs.* control; ^*^P < 0.05 and ^**^P < 0.01 *vs.* C48/80 alone.

MC degranulation can be elicited by the synthetic C48/80, a direct and convenient reagent to study anaphylaxis^32^. We assessed the effect of SHL on C48/80-induced MC degranulation using β-hexosaminidase assay *in vitro*. As shown in Fig. 1, 10 μg/ml of C48/80 evoked a markedly β-hexosaminidase release in the LAD2 cells and rat peritoneal MCs (*P* < 0.01), while SHL potently inhibited the β-hexosaminidase release concentration-dependently (*P* < 0.01) without cytotoxicity (Fig. S1).

Owing to the significant influence of SHL on the allergic mediator release *in vitro*, we next determined the effects of SHL on C48/80-induced anaphylactic shock in mice. As shown in Tables 1 and 2, intraperitoneal injection of C48/80 at 8 mg/kg caused a fatal anaphylactic shock with the mortality of 100%. In comparison, treatment of SHL either 30 min before or 5 min after C48/80 challenge under 2.5 ml/kg and 5 ml/kg dosages dramatically protected the mice against the anaphylactic shock and greatly reduced the mortality, showing the preventive and therapeutic effects of SHL on C48/80-induced anaphylactic shock *in vivo*.

### SHL suppresses IgE-FcεRI-mediated MC activation *in vitro* and *in vivo*

**Figure 2:**
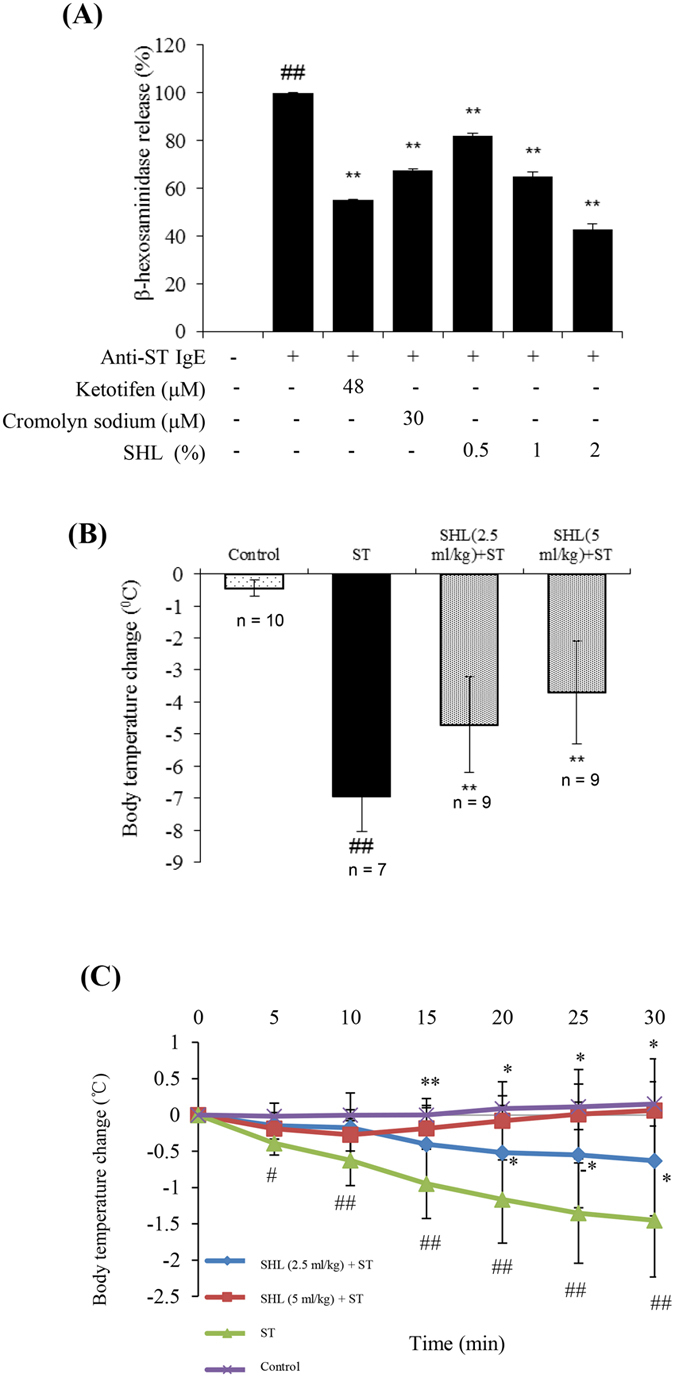
SHL suppressed IgE-FcεRI-mediated MC activation *in vitro* and *in vivo*. **(A)** SHL suppressed IgE-mediated β-hexosaminidase release in RBL-2H3 cells. The supernatants were assayed 1.5 h after ST challenge in the IgE-sensitized RBL-2H3 cells. Ketotifen and cromolyn sodium were used as a positive control. **(B)** Effects of SHL on the body temperature of ST-induced ASA mice. Rectal temperature was measured 30 min after ST challenge. **(C)** Effects of SHL on the body temperature of PSA mice (n = 6). ^#^*P* < 0.05, ^##^*P* < 0.01 *vs.* control group; *P < 0.05, **P < 0.01 *vs.* ST group.

Except for C48/80-induced anaphylactoid reaction, IgE-FcεRI-mediated allergic reactions are another kind of anaphylaxis^33^. Due to the surface expression of the high-affinity FcεRI receptor for IgE and the release of chemical mediators after crosslinking^34^, the sensitized RBL-2H3 cells were used to assess the effect of SHL on the shrimp tropomyosin (ST)-induced degranulation. Our data showed that pretreatment with SHL concentration-dependently dampened IgE-FcεRI-mediated β-hexosaminidase release (Fig. 2A).

We next determined the effect of SHL on ST-induced active systemic anaphylaxis (ASA) in mice. As shown in Fig. 2B, robust hypothermia was observed after ST challenge (ΔT ≈ −7 °C) compared with the normal control group, while pretreatment with SHL significantly attenuated the body temperature decrease (*P* < 0.01). Next, the effect of SHL on passive systemic anaphylaxis (PSA) was shown in Fig. 2C and Fig. S2, after ST challenge, the body temperature of the sensitized mice gradually decreased about 1.5 °C within 30 min, while SHL markedly prevented the body temperature decrease.

### SHL stabilizes MCs by decreasing Ca^2+^_[c]_ levels of resting cells

**Figure 3:**
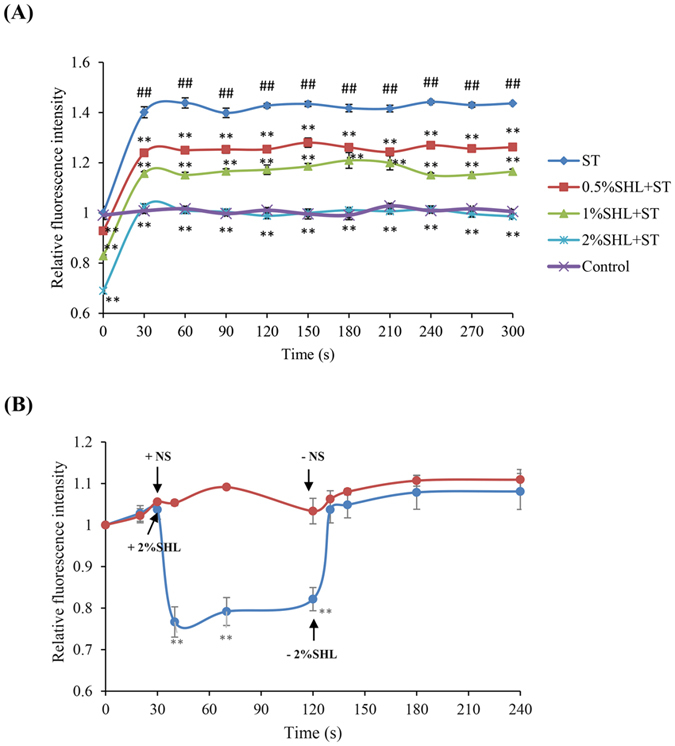
SHL reduced Ca^2+^_[c]_ levels. **(A)** Effect of SHL on IgE-mediated Ca^2+^_[c]_ level in the sensitized RBL-2H3 cells. The sensitized cells were loaded with fluo-3 AM (4 μM) at 30 °C for 30 min. The stained cells were treated with or without SHL for 30 min and then exposed to ST (20 ng/ml). The fluorescent intensity (λ_ex_ 485 nm and λ_em_ 538 nm) was recorded every 30 s. ^##^*P* < 0.01 *vs.* control; ^*^P < 0.05, ^**^P < 0.01 *vs.* ST alone. **(B)** SHL reduced Ca^2+^_[c]_ levels in the resting RBL-2H3 cells. The cells were loaded with fluo-3 AM (4 μM) at 30 °C for 30 min. The stained cells were treated with or without SHL and the fluorescent intensity (λ_ex_ 485 nm and λ_em_ 538 nm) was immediately recorded. ^**^P < 0.01 *vs.* control.

The above findings confirm that SHL dampens C48/80-MrgprX2 and IgE-FcεRI mediated MC degranulation^9^,^35^, both of which depends on the increase of Ca^2+^_[c]_ concentration. Thus, we next investigated whether SHL could affect the Ca^2+^_[c]_ level. As expected, ST challenge markedly elevated Ca^2+^_[c]_ level in the sensitized RBL-2H3 cells, while pretreatment with SHL significantly reduced Ca^2+^_[c]_ level in a concentration-dependent manner (Fig. 3A), without a direct chelation (data not shown). Of note, the Ca^2+^_[c]_ levels before ST challenge (at 0 min) had been significantly reduced in response to pretreatment with SHL compared with the control (Fig. 3A), strongly suggesting that SHL decreased Ca^2+^_[c]_ concentration before the IgE receptor cross-linking. To confirm this, we further measured the effects of SHL on the Ca^2+^_[c]_ level in the resting RBL-2H3 cells. As shown in Fig. 3B, the Ca^2+^_[c]_ level decreased rapidly and sustainably after SHL treatment, and then returned to homeostasis when SHL was withdrawn. Similar effect was observed in both human (LAD2) and mouse (P815) MCs (data not shown). In contrast, cromolyn sodium and ketotifen did not affect Ca^2+^_[c]_ in the resting cells (data not shown) at their effective concentrations on MC degranulation (Figs 1 and 2A).

### SHL reduces Ca^2+^_[c]_ levels via enhancing the Ca^2+^_[m]_ uptake

**Figure 4:**
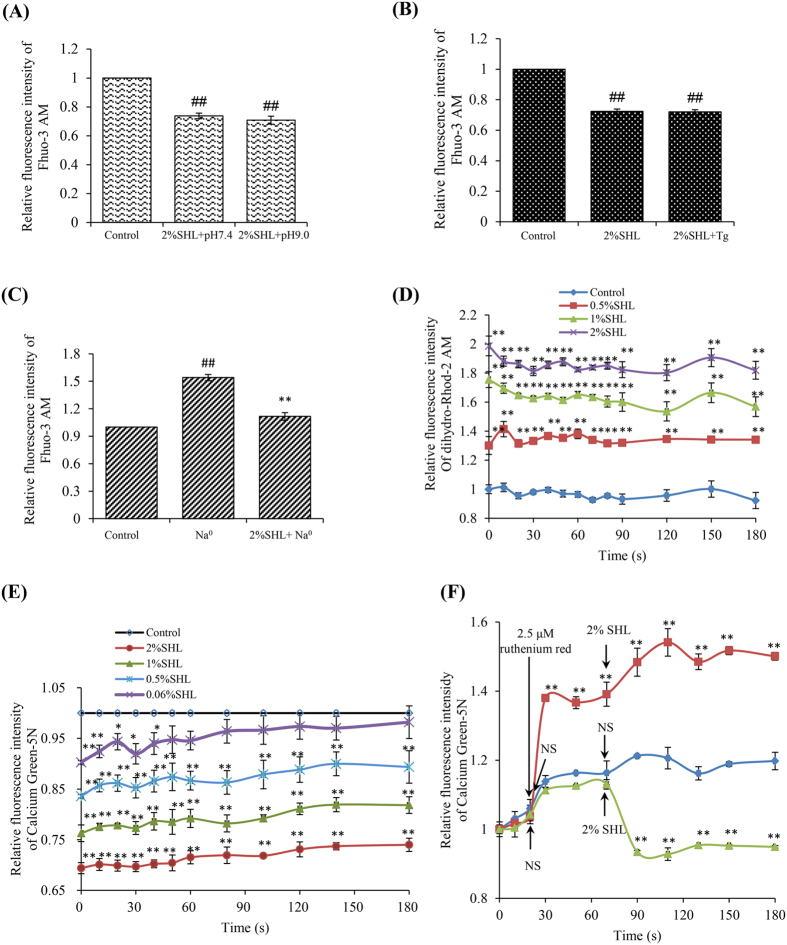
SHL decreased Ca^2+^_[c]_ levels mainly through increasing the Ca^2+^_[m]_ uptake. **(A)** SHL did not affect the activity of PMCA. The effect of SHL on Ca^2+^_[c]_ was determined by a shift to alkaline pH 9.0. ^##^*P* < 0.01 *vs.* control. **(B)** SHL did not affect the activity of SERCA. The effect of SHL on Ca^2+^_[c]_ was determined in the present of thapsigargin (Tg, 5 μm), an inhibitor of the ER Ca^2+^-ATPase. ^##^*P* < 0.01 *vs.* control. **(C)** Effect of SHL on NCX. The effect of SHL on Ca^2+^_[c]_ was measured in a Na^+^-free solution (Na^0^) containing 40 mM KCl. ^##^*P* < 0.01 *vs.* control; ^**^P < 0.01 *vs.* Na^0^. **(D)** SHL concentration-dependently increased Ca^2+^_[m]_ level in RBL-2H3 cells. The cells were loaded with 2 μM of dihydro-rhod-2/AM at 37 °C for 1 h and kept in primary culture for an additional 16 h. The fluorescence intensity of Rhod 2 was determined at λ_ex_ 535 nm and λ_em_ 590 nm. **(E)** SHL increased Ca^2+^_[m]_ uptake in the isolated mouse liver mitochondria. Ca^2+^_[m]_ uptake in isolated mouse liver mitochondria was measured with Calcium Green-5N. **(F)** The increased effects of SHL on Ca^2+^_[m]_ uptake can be blocked by ruthenium red. ^*^P < 0.05 and ^**^P < 0.01 *vs.* control.

The rapid and reversible effect of SHL on Ca^2+^_[c]_ strongly suggested an underlying non-genomic mechanism. To our knowledge, two ways are recognized to reduce Ca^2+^ from the cytosol: extruding of Ca^2+^ through Na^+^-Ca^2+^ exchangers (NCX) and plasma membrane Ca^2+^-ATPase (PMCA), and (or) clearance of Ca^2+^ by resequestration into the ER and mitochondria^36^. Our findings showed that under either inhibition of PMCA activity by alkaline pH 9.0^37^ or suppression of sarco/endoplasmic Ca^2+^-ATPase (SERCA) by thapsigargin^38^, the reduction of SHL on Ca^2+^_[c]_ was not affected (Fig. 4A,B). Unexpectedly, SHL was still able to lower Ca^2+^_[c]_ when the extracellular Na^+^ was withdrawn^39^ (Fig. 4C), seemingly suggesting that SHL inhibited rather than activated NCX. But anyhow, the reduction of Ca^2+^_[c]_ by SHL in the resting cells is independent of PMCA, NCX and SERCA. Then, we found that SHL significantly enhanced the Ca^2+^_[m]_ uptake in a concentration dependent manner (Fig. 4D). By using Calcium Green-5N, Ca^2+^_[m]_ uptake was evaluated in the isolated mouse liver mitochondria, which is of advantage that Ca^2+^ uptake phenotypes can be directly attributed to mitochondria. In agreement with the results in Fig. 4C, SHL (≥0.06%) treatment led to a significant increase of Ca^2+^_[m]_ uptake in response to extramitochondrial pulses of 50 μM of Ca^2+^ (Fig. 4E), which can be blocked by a MCU inhibitor ruthenium red^15^ (Fig. 4F). These results demonstrate that SHL decreases Ca^2+^_[c]_ levels mainly through enhancing the Ca^2+^_[m]_ uptake.

### SHL enhances Ca^2+^_[m]_ uptake by activating MCU

**Figure 5:**
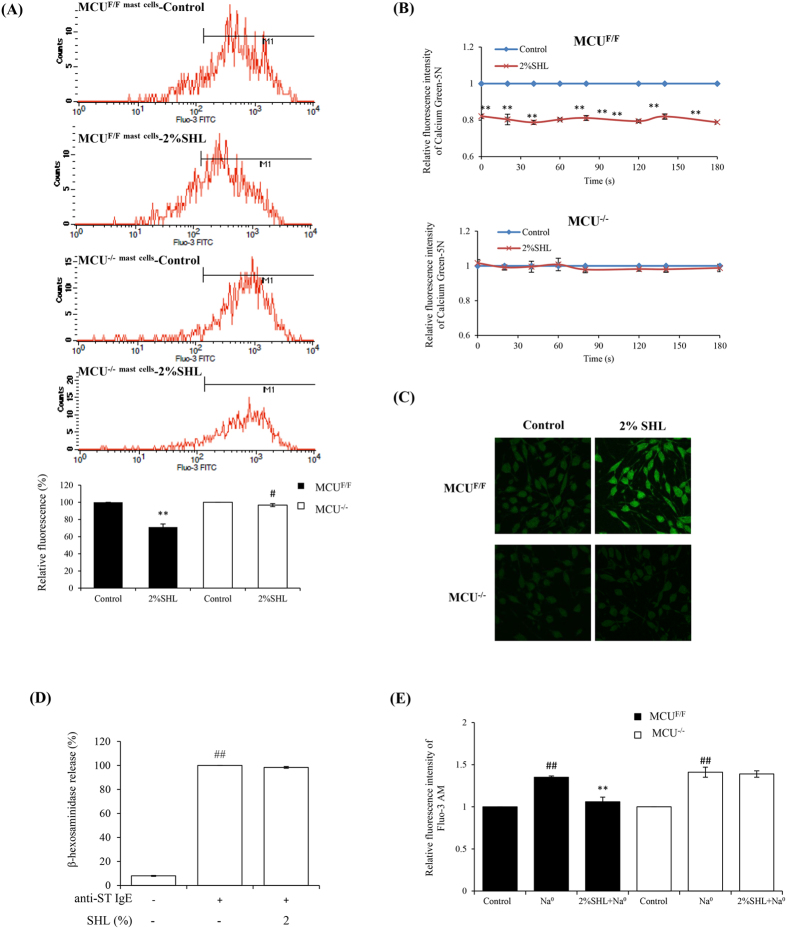
SHL increased Ca^2+^_[m]_ uptake by activating MCU. **(A)** The influence of SHL on Ca^2+^_[c]_ was weakened upon silencing of MCU in mouse peritoneal MCs (n = 3). The cells in the mouse abdominal cavity were loaded with fluo-3/AM and stained with PerCP-eFluor 710 labeled anti-mouse FcεR1 antibody. The fluorescence intensity for the Ca^2+^_[c]_ of MCs were analyzed by a flow cytometer. ^**^P < 0.01 *vs.* Control of MCU^F/F^; ^#^*P* < 0.05 *vs.* Control of MCU^−/−^. **(B)** The influence of SHL on Ca^2+^_[m]_ was completely lost in the liver mitochondria from MCU^−/−^ mice (n = 3). Ca^2+^ uptake in liver mitochondria following the addition of 50 μM CaCl_2_ was measured with Calcium Green-5N. ^**^P < 0.01 *vs.* Control. **(C)** Representative images of SHL-mediated Ca^2+^_[m]_ uptake in MCU^−/−^ RBL-2H3 cells and its negative control cells MCU^F/F^. The Cells were infected with Ad.m-GCaMP2. 24 h later, the fluorescence signal in the mitochondria was captured by confocal microscopy using a 100× oil objective. **(D)** SHL did not suppress IgE-mediated β-hexosaminidase release in MCU^−/−^ RBL-2H3 cells. **(E)** Effect of SHL on NCX in MCU^−/−^ RBL-2H3 cells. ^##^*P* < 0.01 *vs.* control; ^**^P < 0.01 *vs.* Na^0^.

Calcium transport between the cytoplasm and the mitochondrial matrix involves the passage of Ca^2+^ across both the outer and inner mitochondrial membranes (OMM and IMM). The overall permeability of the OMM for Ca^2+^ is relatively high, while the IMM presents a tight barrier for Ca^2+^ 40. Early studies have revealed that MCU protein, which can form a Ca^2+^ channel in lipid bilayer in the IMM, forms the basis of the primary mechanism for Ca^2+^_[m]_ transport^14^,^15^,^41^. Moreover, our above result (Fig. 4F) also showed that the effect of SHL on Ca^2+^_[m]_ uptake can be completely blocked by ruthenium red, highly implicating that SHL enhanced Ca^2+^_[m]_ uptake might through activating MCU. To verify whether MCU is indeed involved in the effect of SHL on Ca^2+^_[m]_, we silenced MCU in mice using a Entranster TM *in vivo* transfection reagent. The resulting mice, termed MCU^−/−^ mice, lack MCU protein in peritoneal MCs and liver mitochondria compared with the negative control mice (MCU^F/F^). The fluorescence intensity for the Ca^2+^_[c]_ of FcεRI^+^ cells, namely MCs, was analyzed by a flow cytometer (FACSCalibur, BD, USA). It was found that SHL potently reduced Ca^2+^_[c]_ levels of peritoneal MCs in MCU^F/F^ mouse with a decreased percentage of 29%, while this effect was notably weakened in the MCU^−/−^ cells with only a decreased percentage of 3.3% (Fig. 5A).

In the isolated liver mitochondria from the MCU^F/F^ and MCU^−/−^ mice, the activation of SHL on the Ca^2+^_[m]_ uptake was completely lost upon silencing of MCU (Fig. 5B). Moreover, in the MCU^−/−^ RBL-2H3 cells, the effect of SHL on the Ca^2+^_[m]_ uptake and MC degranulation also disappeared (Fig. 5C,D). It was in this MCU defective cells that we did not observe the effect of SHL in Fig. 4C (Fig. 5E), indicating that SHL did not affect NCX. Taken together, these findings reveal that SHL increases Ca^2+^_[m]_ uptake through activating MCU to decrease Ca^2+^_[c]_ level, thus dampens MC degranulation.

### Quercetin, caffeic acid, ursolic acid, D-(-)-quinic acid and methyl salicylate lower Ca^2+^_[c]_ levels of resting cells

**Figure 6:**
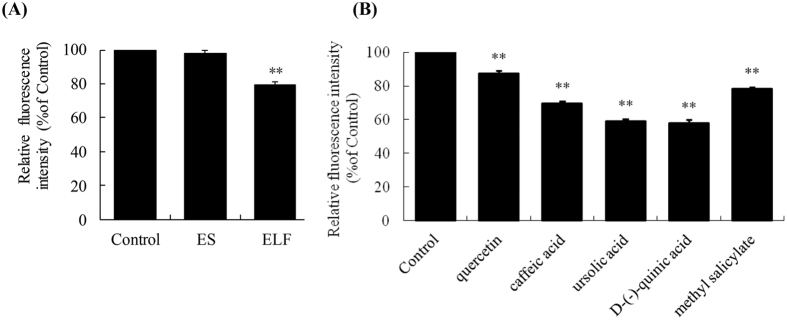
Effects of the active fractions and constituents in SHL on Ca^2+^_[c]_ levels in the resting RBL-2H3 cells. **(A)** Effects of ES and ELF on Ca^2+^_[c]_ levels in the resting RBL-2H3 cells. The final concentrations of ES and ELF are 192.4 μg/ml and 660 μg/ml, which are equivalent to the concentration in 2% SHL injection. **(B)** Effects of quercetin, caffeic acid, ursolic acid, D-(-)-quinic acid and methyl salicylate (10 μg/ml) in ELF on Ca^2+^_[c]_ levels in RBL-2H3 cells. Ca^2+^_[c]_ levels were expressed as the relative fluorescence intensity, and the values in control cells were arbitrarily normalized as 100%. ^**^P < 0.01 *vs.* control.

According to the Chinese Pharmacopoeia^23^, SHL is a mixture of the extract of *Scutellariae Radix* (ES) and the extract of *Lonicerae Japonicae Flos* and *Fructus Forsythiae* (ELF). Thus, to identify the active constituents in SHL, we first evaluated the effects of ES and ELF at the equivalent concentrations in 2% SHL on Ca^2+^_[c]_ in the resting RBL-2H3 cells. As shown in Fig. 6A, ELF, rather than ES, significantly reduced Ca^2+^_[c]_ levels compared with untreated control in a nontoxic manner (data not shown). Next, we tested the effects of 26 constituents from ELF (Table S1) on the Ca^2+^_[c]_ levels. Figure 6B shows that Ca^2+^_[c]_ levels were significantly decreased in the presence of quercetin, caffeic acid, ursolic acid, D-(-)-quinic acid and methyl salicylate at 10 μg/ml in a nontoxic manner (data not shown), and the fore 3 constituents could be detected by the HPLC-UV according to the Chinese Pharmacopoeia^23^ (Fig. S3), suggesting that they might be the major active constituents of SHL.

## Discussion

MCs are key effector cells that can act as potent initiators and amplifiers in allergy, immunity, and inflammation by secreting multiple mediators^6^,^42^. Our findings demonstrated that SHL markedly dampened C48/80- and IgE-mediated MC degranulation *in vitro* and *in vivo* (Figs 1 and 2 and Tables 1 and 2), showing an impressive influence on the MC activation. Further study indicated that SHL stabilized MC via a rapid, potent and reversible effect on Ca^2+^_[c]_ level of resting cells (Fig. 3B), which is different from the conventional MC stabilizers (e.g., cromolyn sodium and ketotifen).

As a MC activator, C48/80 could induce a rapid release of allergic mediators and consequently lead to a systemic fatal anaphylaxis^43^,^44^. In accordance with previous studies^45^,^46^,^47^, intraperitoneal injection of C48/80 (8 mg/kg) induced a fatal anaphylactic shock with a mortality of 100% within 1 h. Unexpectedly but excitedly, by a single intraperitoneal treatment with 3.34 times adult oral dosage of SHL (5 ml/kg) or 600 times that of ketotifen (47 μmol/kg), the survival rate of SHL group were actually far more than that of ketotifen group (Tables 1 and 2).

At present, the commonly-used allergen in the IgE-FcεRI-mediated allergy research is ovalbumin (OVA) to mimic type I hypersensitivity^48^,^49^. However, our previous result showed that the sensibility of common mice response to OVA was not satisfactory, especially in the absence of an adjuvant (data not shown), which might be associated with the immune tolerance induced by a long-term consumption of eggs powder in rodents’ fodder^50^. As we known, seafood allergy is widely recognized as a universal health care issue^51^,^52^,^53^ and is one of the most common forms of food allergies^54^,^55^. Shrimp protein is a major allergen in the shellfish-induced allergy study^56^,^57^. Thus, we extracted a purified ST (Fig. S4) from the *Metapenaeus ensis* by isoelectric precipitation^56^. Satisfactorily, compared with OVA, ST dramatically elevated the total IgE level in the mouse sera, showing a more sensitive responsivity (data not shown). Therefore, ST instead of OVA was used in our study. In the IgE-FcεRI-mediated β-hexosaminidase release (*in vitro*) and PSA (*in vivo*), SHL exerted markedly anti-anaphylactic effects (Fig. 2A,C). In ASA mice, SHL also significantly attenuated the body temperature decrease (Fig. 2B). In particular, hypothermia in ASA mice was far more intense than that in PSA mice, and SHL exerted more effective protection on PSA than ASA (Fig. 2B), which may be attributed to the fact that ASA is mediated not only by IgE, but also by IgG^58^.

Theoretically, it is feasible for a drug to stabilize MCs through buffering the Ca^2+^_[c]_ rises via accumulating Ca^2+^_[c]_ into mitochondrial matrix. However, difficulties lie in the experimental practices. To our knowledge, MCU mediates Ca^2+^ uptake into the matrix to regulate metabolism, cytoplasmic Ca^2+^ signaling and cell death^59^. The uptake is electrogenic, driven by the large voltage present across the IMM (ΔΨ m) developed by proton pumping by the respiratory chain^21^,^60^. Balanced Ca^2+^_[m]_ is critical for the regulation of mitochondrial functions such as fission, fusion and ATP production^61^. On one hand, Ca^2+^_[m]_ rise is the stimulation of Ca^2+^-sensitive dehydrogenases of the Krebs cycle, tuning ATP synthesis to the increased needs of a cell; on the other hand, uncontrolled Ca^2+^_[m]_ overload can lead to the opening of the mitochondrial permeability transition pore with disruption of mitochondrial membrane potential (MMP)^62^. Excess Ca^2+^ entry in mitochondria has been associated with apoptosis and necrosis in many pathological states^63^. Most recently, Vais and his colleagues found that mitochondria were protected from Ca^2+^ depletion and overload by a unique complex involving Ca^2+^ sensors on both sides of the IMM, coupled through EMRE^59^. Obviously, the dynamic regulation of Ca^2+^_[m]_ is a highly sophisticated process. Thus, as a MC stabilizer through enhancing Ca^2+^_[m]_ uptake, how to strike a better balance between the effectivity and toxicity is a serious challenge. Our findings indicate that it is through activating MCU that SHL, which has been using for the allergic diseases clinically, decreases Ca^2+^_[c]_ level to stabilize MCs. Both the effectivity and safety (non-toxic) of SHL are compatible *in vitro* and *in vivo*, indicating that the Ca^2+^_[m]_ increase induced by SHL through activating MCU is sustainable to a certain degree. Of course, the pharmacological reversibility of SHL is also an essential factor. Moreover, excess Ca^2+^ entry in mitochondria (Ca^2+^_[m]_ overload) causes more reactive oxygen species (ROS) generation, a by-product of Krebs cycle, whose elevation is a key event that leads to further organelle depolarization and loss of MMP, thus resulting in a vicious cycle^64^,^65^. Perhaps not by coincidence, SHL possesses scavenging effect on the excess intracellular ROS thus protecting MMP^25^, which may also play an important role for striking the balance between effectivity and non-toxic.

It is generally recognized that MCU is a Ca^2+^-activated Ca^2+^ channel whose activation depends on the increase of Ca^2+^_[c]_ concentration^15^. But, unlike the known MCU agonist histamine^15^, SHL can activate MCU independent of Ca^2+^_[c]_ rise. Thus, we were able to observe that SHL rapidly reduced Ca^2+^_[c]_ levels in the resting cells (Fig. 3B) and enhanced Ca^2+^_[m]_ uptake in the isolated liver mitochondria (Fig. 4E), suggesting that the active constituents in SHL (e.g., quercetin, caffeic acid, ursolic acid, etc.) can rapidly enter into the cells to directly act on the mitochondria to active MCU. It is noteworthy that although five constituents reduced Ca^2+^_[c]_ of resting cells (Fig. 6B), their effective concentrations (10 μg/ml) on Ca^2+^_[c]_ was far higher than their equivalent concentrations in 2% SHL, indicating that there might be a series of active ingredients similar to these five constituents to collectively act on MCU to markedly stabilize MCs.

In summary, SHL is a potent inhibitor of MC activation through decreasing Ca^2+^_[c]_ level by activating MCU. By virtue of the effect on resting Ca^2+^_[c]_, the degree of MC activation was potently suppressed, which not only could limit allergic disease, but also might be beneficial to some non-allergic diseases involved MC activation, such as atherosclerosis^66^, obesity^67^,^68^, diabetes^67^,^68^, chronic obstructive pulmonary disease^69^, cancer^70^, postoperative ileus^71^ and fibromyalgia^72^, etc. However, our finding, together with the fact that SHL has already been used in the clinic for decades, may offer a suitable novel target for the clinical management of aberrant MC activation in diseases.

## Methods

### Materials

SHL injection and its 2 intermediate fractions (ES and ELF), which were prepared according to the Chinese Pharmacopoeia^23^ were from Duoduo Pharmaceutical Co., Ltd. (Jiamusi, Heilongjiang, China). C48/80, 4-Methylumbelliferyl N-acetyl-β-D-glucosaminide, and Pluonic F-127 were purchased from Sigma-Aldrich (St Louis, MO, USA). Fluo-3 AM Ester and rhod-2/AM were from Biotium (San Francisco, CA, USA). Ketotifen and cromolyn sodium were from TCI (Tokyo, Japan) and National Institutes for Food and Drug Control (Beijing, China), respectively. Calcium Green-5N and PerCP-eFluor 710 labeled anti-mouse FcεR1 antibody were obtained from Invitrogen (Carlsbad, CA, USA) and eBioscience (San Diego, CA, USA), respectively. The transfection reagents Entranster TM-*in vivo* and -H4000 were from Engreen Biosystem (Beijing, China). Balb/c mice (male, 18–20 g) and SD rats (male, 160–180 g) were from Vital River Experimental Animal Services (Beijing, China).

### siRNA and plasmid

The MCU siRNA and their negative controls were synthesized by GenePharma Co., Ltd. (Shanghai, China). The plasmid pcDNA3.1-mito-GCaMP2 was a kind gift from Dr. Xianhua Wang (Institute of Molecular Medicine, Peking University, Beijing, China). In this plasmid, the GCaMP2 calcium indicator was ligated with a mitochondrial targeting sequence^73^.

### Cells

Rat basophilic leukemia cell line (RBL-2H3) and mouse mastocytoma cell line (P815) were purchased from the cell bank of Chinese Academy of Sciences (Shanghai, China). Human LAD2 cell line (from Michael D. Gershon, MD, Columbia University, USA) was presented as a gift from Prof. Renshan Sun (the Third Military Medical University, Chongqing, China). Peritoneal MCs were isolated from SD rats.

### Isolation of ST

ST from the *Metapenaeus ensis* was extracted and purified by an isoelectric precipitation method as previously described^56^. Protein content of the purified fraction was assayed by Bradford method^74^, and the purity of the obtained ST was >98% (Fig. S4).

### Production of mouse anti-ST monoclonal IgE

The preparation of antibody was similar to our previously described method^75^ except for the substitution of Freund’s adjuvant for Imject Alum.

### β-hexosaminidase release assay

The β-hexosaminidase release assay was performed as previously described with some modifications^76^. For the measurements of IgE-induced β-hexosaminidase release, RBL-2H3 cells were seeded in the 48-well plates at 5.0 × 10^5^ cells/well and sensitized with anti-ST monoclonal IgE (25 μg/ml) at 37 °C overnight. The cells were washed by Hank’s balanced salt solution (HBSS) supplemented with 0.1% (w/v) BSA and pre-incubated with SHL in 120 μl of HBSS at 37 °C for 30 min followed by adding 20 ng/ml of ST for further 1.5 h incubation. 30 μl of the supernatant was transferred to a 96-well black flat-bottom plate accompany with 50 μl of substrate solution (0.57 mg/ml 4-Methylumbelliferyl N-acetyl-β-D-glucosaminide in the buffer contained 133 mM sodium citrate and 133 mM NaCl, pH 4.3). The reaction proceeded at 37 °C for 1.5 h and was stopped by adding stop buffer (50 mM glycine and 5 mM EDTA-Na_2_, pH 10.5; 200 μl/well). Fluorescence was determined with a fluorescence microplate reader at λ_ex_ 355 nm and λ_em_ 460 nm.

For the measurements of C48/80-induced β-hexosaminidase release, the LAD2 cells (6.0 × 10^4^ cells/well) or the peritoneal MCs (1.0 × 10^6^ cells/well) were seeded in the 96-well plates and pretreated with SHL at 37 °C for 30 min followed by adding C48/80 (10 μg/ml) for further 1.5 h incubation. β-hexosaminidase in the supernatant was determined.

### C48/80-induced anaphylactic shock in mice

Balb/c mice were kept in standard laboratory conditions of temperature and humidity with a 12 h light/dark cycle. All experiments were carried out according to the National Institutes of Health Guide for Care and Use of Laboratory Animals and were approved by the Animals Ethics Committee of the Institute of Medicinal Plant Development of the Chinese Academy of Medical Sciences. The mice were given the intraperitoneal injection of C48/80 at 8 mg/kg^77^. For the preventive effect, SHL or the positive controls (ketotifen or cromolyn sodium) was i.p. injected only once 30 min before C48/80 administration (designated “single treatment”). For the therapeutic effect, SHL or the positive controls was i.p. injected only once 5 min after C48/80 challenge. Mortality was monitored for 1 h after induction of anaphylactic shock.

### PSA

Mice were passively sensitized intravenously (i.v.) with 40 μg/mouse of anti-ST monoclonal IgE, while the control group were given the equal volume of physiologic saline. 24 h later, the mice were challenged (i.v.) with 20 μg/mouse of ST after pretreatment with SHL or physiologic saline (i.p.) for 20 min. The rectal temperature was measured by a thermal probe (ChengDu Instrument Factory, China) for 30 min using a polygraph (RM6240, Chengdu, China).

### ASA

Mice received an i.p. injection of 100 μl of Imject Alum containing 60 μg/mouse ST and were immunized again 7 days later. 2 days after the second immunization, the mice were pretreated with SHL (2.5 ml/kg or 5 ml/kg) or physiologic saline (Control group and ST model group) for 30 min and then challenged by a rapid intravenous infusion (via the lateral tail vein) of 5 μg/mouse of ST. The mice in the control group were received the same solution without ST. To monitor changes in body temperature associated with anaphylaxis, rectal temperature was measured 30 min after ST challenge.

### Measurement of Ca^2+^_[c]_ level

Measurement of the Ca^2+^_[c]_ level was performed using the calcium-reactive fluorescence probe Fluo-3/AM as previously described with slight modifications^78^. Briefly, the cells were resuspended (2 × 10^6^ cells/ml) and incubated for 30 min in the dark at 30 °C with Fluo-3/AM (4 μM) in the presence of 0.04% (w/v) Pluonic F-127 in HEPES buffer (10 mM HEPES, 135 mM NaCl, 1 mM Na_2_HPO_4_, 1 mM CaCl_2_, 5 mM KCl, 0.5 mM MgCl_2_, 5 mM glucose and 0.1%BSA, pH 7.4). 4 mM probenecid was added to avoid leakage of fluo-3. After removing the dye, the cells were treated with SHL or normal saline (the control group) and the fluorescent intensity was immediately determined at λ_ex_ 485 nm and λ_em_ 538 nm using a spectrofluorimeter (Thermo Electron, Washington, USA).

To assay the Ca^2+^_[c]_ levels in mouse peritoneal MCs, the cells in the mouse abdominal cavity were isolated and loaded with Fluo-3/AM (4 μM) at 30 °C for 30 min. MCs could be recognized and analyzed by a FACSCalibur flow cytometer after staining with PerCP-eFluor 710 labeled anti-mouse FcεR1 antibody at 25 °C for 15 min.

### Measurement of Ca^2+^_[m]_ uptake

Measurement of Ca^2+^_[m]_ level in RBL-2H3 cells was performed using the mitochondrially localizing Ca^2+^-reactive fluorescence probe, rhod-2/AM, as previously described^79^. To improve the discrimination between cytosolic and mitochondrially localized dye, 5 μM rhod-2/AM was reduced to the colorless, nonfluorescent dihydro-rhod-2/AM by sodium borohydride, according to the manufacturer’s protocol. RBL-2H3 cells were loaded with dihydro-rhod-2/AM (2 μM) at 37 °C for 1 h. The residual cytosolic fraction of the dye was eliminated when the cells were kept in primary culture for an additional 16 h after loading, whereas the mitochondrial dye fluorescence was maintained. The fluorescence intensity of rhod-2 was determined at λ_ex_ 535 nm and λ_em_ 590 nm.

Mouse liver mitochondria were isolated and further purified^80^. Ca^2+^_[m]_ uptake was measured using Calcium Green-5N according to previously described^14^.

The recombinant adenovirus (Ad.m-GCaMP2) based on the pcDNA3.1-mito-GCaMP2 was produced by Hanbio Biotechnology Co., Ltd. (Shanghai, China). Cells were infected with Ad.m-GCaMP2. 24 h later, the fluorescence signal in the mitochondria was captured by confocal microscopy (Fluoview FV1000, Olympus, Japan) using a 100× oil objective.

### MCU siRNA transfected *in vivo* and *in vitro*

The mice were injected via tail vein with MCU siRNA (siRNA-MCU1#: sense 5′-GCG CCA GGA AUA UGU UUA UTT-3′ and antisense 5′-AUA AAC AUA UUC CUG GCG CTT-3′; siRNA-MCU2#: sense 5′-CCA AAG AGA GAC CUC CUA ATT-3′ and antisense 5′-UUA GGA GGU CUC UCU UUG GTT-3′) or the negative control siRNA (sense 5′-UUC UCC GAA CGU GUC ACG UTT-3′ and antisense 5′-ACG UGA CAC GUU CGG AGA ATT-3′) on days 1 and 4 (3 OD/mouse). Entranster TM-*in vivo* transfection reagent was used to deliver the siRNA according to the manufacturer′s recommendations. On day 5, the mice were sacrificed after anesthetization. Fresh liver and the peritoneal MCs were immediately isolated for the assay.

The rat MCU siRNA (siRNA-MCU1#: sense 5′-GCC AGA GAC AGA CAA UAC UTT-3′ and antisense 5′-AGU AUU GUC UGU CUC UGG CTT-3′; siRNA-MCU2#: sense 5′-GGA GAA GGU ACG GAU UGA ATT-3′ and antisense 5′-UUC AAU CCG UAC CUU CUC CTT-3′) or the negative control (sense 5′-UUC UCC GAA CGU GUC ACG UTT-3′ and antisense 5′-ACG UGA CAC GUU CGG AGA ATT-3′) was transfected into RBL-2H3 cells using Entranster TM-H4000 as described in the manufacturer’s protocol.

### Statistical analysis

Data represent the mean ± SD of at least three independent experiments. Statistical analysis was performed by one-way ANOVA. A student’s *t* test was used when only two groups were compared. The difference was considered to be statistically significant when *P* < 0.05.

## Additional Information

**How to cite this article:** Gao, Y. *et al*. The Three-Herb Formula Shuang-Huang-Lian stabilizes mast cells through activation of mitochondrial calcium uniporter. *Sci. Rep.* **7**, 38736; doi: 10.1038/srep38736 (2017).

**Publisher's note:** Springer Nature remains neutral with regard to jurisdictional claims in published maps and institutional affiliations.

## Supplementary Material

Supplemental Material

## Figures and Tables

**Table 1:** Preventive effect of SHL on C48/80-induced anaphylactic shock in mice (n = 12).

Group	Dose	Survival rate (%)
Control	None (saline)	0
Ketotifen*	47 μmol/kg	41.7
Cromolyn sodium*	0.4 mmol/kg	58.5
SHL*	1.25 ml/kg	16.7
SHL	2.5 ml/kg	75
SHL	5 ml/kg	100

^*^SHL or the positive control (ketotifen or cromolyn sodium) was i.p. injected only once 30 min before the C48/80 administration.

**Table 2:** Therapeutic effect of SHL on C48/80-induced anaphylactic shock in mice (n = 12).

Group	Dose	Survival rate (%)
Control	None (saline)	0
Ketotifen*	47 μmol/kg	25
Cromolyn sodium*	0.4 mmol/kg	25
SHL*	1.25 ml/kg	8.3
SHL	2.5 ml/kg	58.3
SHL	5 ml/kg	91.7

^*^SHL or the positive controls (ketotifen or cromolyn sodium) was i.p. injected only once 5 min after the C48/80 challenge.
