# Supplementary material for: The Three-Herb Formula Shuang-Huang-Lian stabilizes mast cells through activation of mitochondrial calcium uniporter
Source: Sci Rep. 2017 Jan 3;7:38736. doi: 10.1038/srep38736 (PMC5206722; doi:10.1038/srep38736)
Supplement: Supplemental Material [file srep38736-s1.pdf]

The Three-Herb Formula Shuang-Huang-Lian stabilizes mast cells through activation of mitochondrial calcium uniporter

Yuan Gao, Rui Hou, Qiaoling Fei, Lei Fang, Yixin Han, Runlan Cai, Cheng Peng* and Yun Qi*

Supplemental material

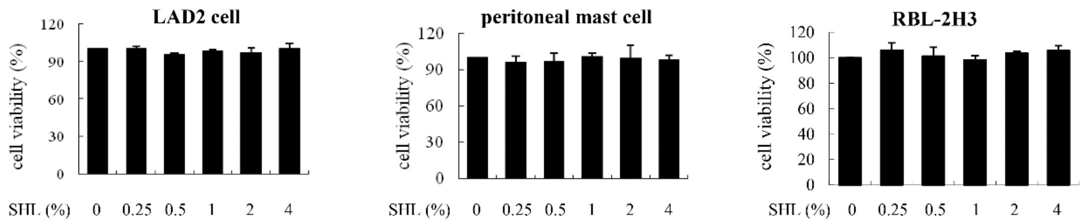

Fig. S1. Effects of SHL on the cells' viability. LAD2 cells, peritoneal MCs or RBL-2H3 cells were incubated with SHL at the indicated concentrations for 24 h and the cell viability was evaluated by MTS assay. Data represents the mean ± SD of three independent experiments.

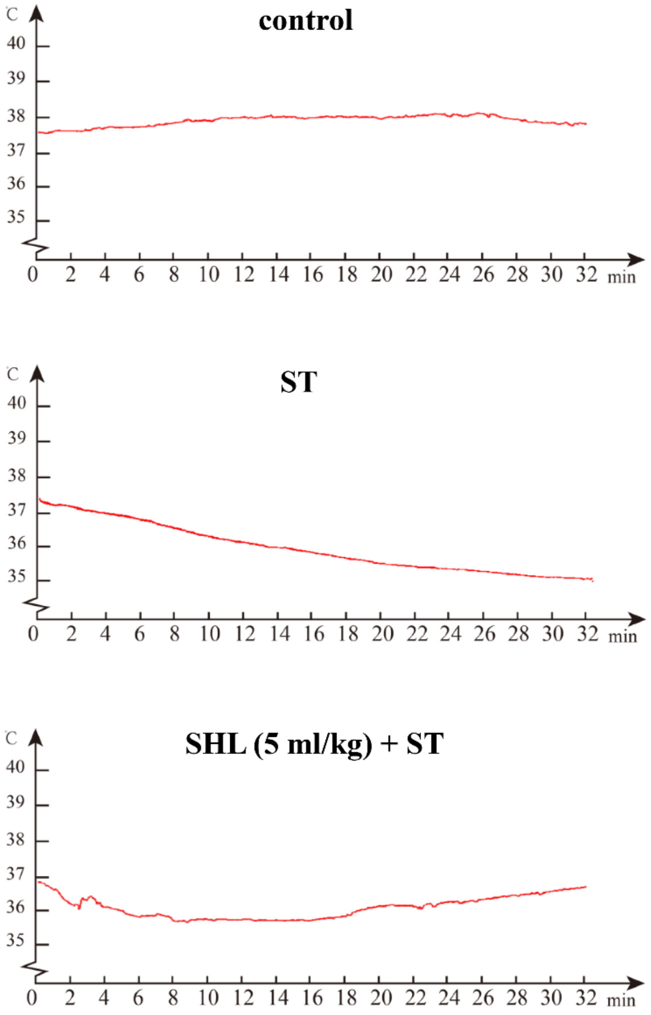

Fig. S2. Typical tracing showing the effect of SHL on the body temperature of PSA mice.

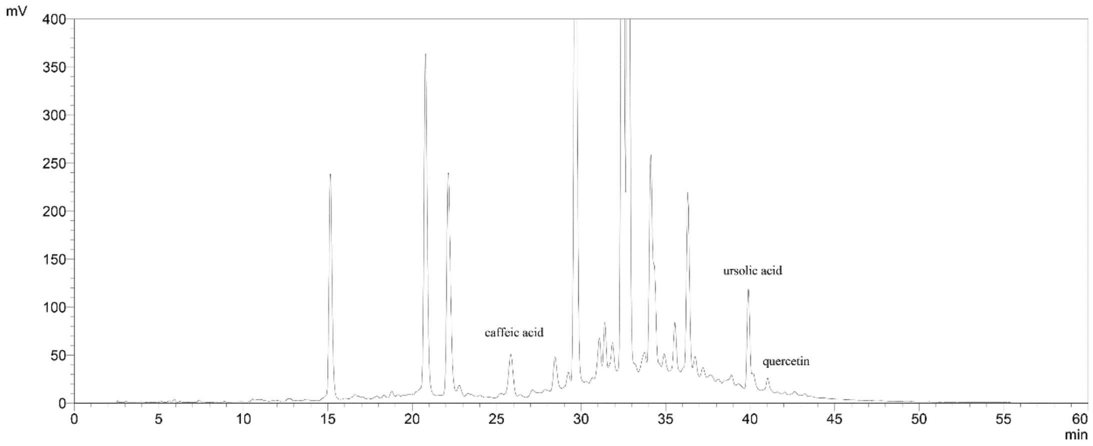

Fig. S3 A representative chromatogram of caffeic acid, ursolic acid and quercetin.

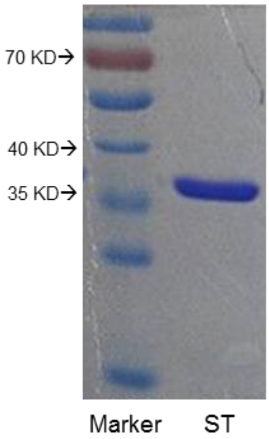

Fig. S4. Analysis of ST extracted from the *Metapenaeus ensis* by SDS-PAGE.

Table S1 Herbal sources of 26 constituents in the ELF.

<table>
<tr><th rowspan="2">No.</th><th rowspan="2">Constituent</th><th colspan="2">Source</th></tr>
<tr><th>Lonicerae Japonicae Flos</th><th>Fructus Forsythiae</th></tr>
<tr><td>1</td><td>neochlorogenic acid</td><td>+</td><td></td></tr>
<tr><td>2</td><td>luteolin</td><td>+</td><td></td></tr>
<tr><td>3</td><td>chrysophanol</td><td colspan="2">+*</td></tr>
<tr><td>4</td><td>oleanolic acid</td><td></td><td>+</td></tr>
<tr><td>5</td><td>isopimpinellin</td><td colspan="2">+*</td></tr>
<tr><td>6</td><td>rutin</td><td>+</td><td>+</td></tr>
<tr><td>7</td><td>cryptochlorogenic acid</td><td>+</td><td></td></tr>
<tr><td>8</td><td>physcion</td><td colspan="2">+*</td></tr>
<tr><td>9</td><td>methyl salicylate</td><td></td><td>+</td></tr>
<tr><td>10</td><td>D-(-)-quinic acid</td><td>+</td><td></td></tr>
<tr><td>11</td><td>isochlorogenic acid A</td><td>+</td><td></td></tr>
<tr><td>12</td><td>isochlorogenic acid B</td><td>+</td><td></td></tr>
<tr><td>13</td><td>isochlorogenic acid C</td><td>+</td><td></td></tr>
<tr><td>14</td><td>quercitrin</td><td>+</td><td></td></tr>
<tr><td>15</td><td>quercetin</td><td>+</td><td>+</td></tr>
<tr><td>16</td><td>chlorogenic acid</td><td>+</td><td></td></tr>
<tr><td>17</td><td>eugenol</td><td>+</td><td></td></tr>
<tr><td>18</td><td>forsythiaside B</td><td></td><td>+</td></tr>
<tr><td>19</td><td>hyperin</td><td>+</td><td></td></tr>
<tr><td>20</td><td>ursolic acid</td><td></td><td>+</td></tr>
<tr><td>21</td><td>caffeic acid</td><td>+</td><td>+</td></tr>
<tr><td>22</td><td>acetophenone</td><td></td><td>+</td></tr>
<tr><td>23</td><td>forsythiaside</td><td></td><td>+</td></tr>
<tr><td>24</td><td>forsythin</td><td></td><td>+</td></tr>
<tr><td>25</td><td>forsythiaside E</td><td></td><td>+</td></tr>
<tr><td>26</td><td>caffeoylquinic acid</td><td>+</td><td></td></tr>
</table>

* These constituents had been identified in the SHL injection, but their herbal sources had not been confirmed.
